# AdipoRon and Pancreatic Ductal Adenocarcinoma: a future perspective in overcoming chemotherapy-induced resistance?

**DOI:** 10.20517/cdr.2022.34

**Published:** 2022-06-21

**Authors:** Luigi Sapio, Angela Ragone, Annamaria Spina, Alessia Salzillo, Silvio Naviglio

**Affiliations:** Department of Precision Medicine, University of Campania “Luigi Vanvitelli”, Naples 80138, Italy.

**Keywords:** PDAC, AdipoRon, gemcitabine, resistance

## Abstract

The latest scientific knowledge has provided additional insights accountable for the worst prognosis for pancreatic ductal adenocarcinoma (PDAC). Among the causative factors, the aptitude to develop resistance towards approved medications denotes the master key for understanding the lack of improvement in PDAC survival over the years. Even though several compounds have achieved encouraging results at preclinical stage, no new adjuvant agents have reached the bedside of PDAC patients lately. The adiponectin receptor agonist AdipoRon is emerging as a promising anticancer drug in different cancer models, particularly in PDAC. Building on the existing findings, we recently reinforced its candidacy in PDAC cells, proposing AdipoRon either as a suitable partner in gemcitabine-based treatment or as an effective drug in resistant cells. Crossing the current state-of-the-art, herein we provide a critical perspective on AdipoRon to figure out whether this receptor agonist can potentially be considered a future therapeutic choice in overcoming chemotherapy-induced resistance, expressly in PDAC.

## INTRODUCTION

Considered as one of the most aggressive malignancies, pancreatic ductal adenocarcinoma (PDAC) accounts for almost all cases of pancreatic cancer^[1]^. Due to the lack of specific and sensitive biomarkers, diagnosis usually happens too late, or rather when the advanced metastatic stage has already occurred^[2]^. The disappointing outcomes provided by the available therapeutic regimes have further contributed to worsening PDAC prognosis, whose five-year survival rate currently stands at 10%^[3]^. Regrettably, according to the existing predictive studies, PDAC will become the second leading cause of cancer death in the next decade.

Even though a combination of surgery and adjuvant chemotherapy represents the approved curative regimen for PDAC management^[4]^, due to the advanced or metastasized stage at diagnosis, not more than 20% of patients can undergo surgical removal^[5]^. Therefore, in both resectable and non-resectable PDAC conditions, the only partially viable therapeutic strategy remains chemotherapy. Consistent with clinical practice guidelines, the first-line treatment involves the use of gemcitabine, which can be administered in combination with albumin-bound paclitaxel or FOLFIRINOX (oxaliplatin, irinotecan, leucovorin, and 5-fluorouracil) treatment cycles^[6]^. Although both combination therapies have moderately improved either overall or median progression-free survival, the highest incidence of mild to severe adverse reactions has strongly restricted their usage in prolonged regimes^[7]^. Therefore, gemcitabine represents the only alternative to palliative care sometimes. Disappointingly, while PDAC cells are initially extremely responsive to this cytotoxic agent, most patients easily develop resistance within a few weeks, thus compromising the success rate of the treatment^[8]^.

Underlying the fully fledged mechanism of chemotherapy resistance is very intricate in cancer, given that many determining factors may be involved. Essentially, both innate and adaptive mechanisms can concurrently lead to the PDAC-resistant phenotype^[9]^. Analogously, native and acquired alterations in either nucleoside transporters or metabolic enzymes may prompt PDAC cells to gemcitabine resistance^[10]^. Due to its hydrophilicity, cellular uptake is mediated by human equilibrative nucleoside transporters (hENTs) and human concentrative nucleoside transporters (hCNTs), although hENT1 accounts for transporting almost the entire amount of gemcitabine into cytosol^[11]^. Once inside, gemcitabine is first phosphorylated by deoxycytidine kinase (dCK), and then converted to nucleoside diphosphate (dFdCDP) and triphosphate (dFdCTP), successively^[12]^. Its metabolic inactivation, instead, is regulated by cytidine deaminase (CDA) or, after phosphorylation, deoxycytidylate deaminase (dCTD)^[13]^. Conversely, gemcitabine can inhibit dCDT activity both directly and through its active metabolite, dFdCTP^[14,15]^*.* Considering the gemcitabine-related metabolism as a whole, it is quite clear that changes in hENT1 levels, as well as CDA dysregulations, can play a decisive role in defining the degree of resistance against this chemotherapy drug. In PDAC, for instance, variations in CDA expression or activity have been correlated with impaired gemcitabine responsiveness^[16-18]^. Although gemcitabine is not considered a canonical target of multidrug resistance-associated proteins (MRPs)^[19,20]^, different studies have displayed a sort of collateral modulation in ATP-binding cassettes (ABCs), expressly in PDAC cells unresponsive to deoxycytidine analog^[13,21-23]^. Besides the tumor-related mechanisms, stroma may further contribute to the establishment of a non-permissive cytotoxic microenvironment^[24]^, both producing a barrier for drug delivery and influencing cancer cell behaviors^[25,26]^. Secreting soluble factors, remodeling extracellular matrix, delivering exosomes, reprogramming the metabolic process, and the epigenetic landscape of tumor cells are some of the stroma-related properties leading to resistance^[27,28]^. In addition, there is an ever-growing awareness of the crucial role driven by cancer cell metabolism in influencing drug response^[9,29]^. In accordance with this perspective, targeting precise metabolic pathways, which include, among others, glycolysis and mitochondrial oxidative phosphorylation (OXPHOS)^[30-32]^, has recently been recognized as a promising pharmacological approach to overcome chemoresistance in PDAC.

Starting from the adiponectin (Acrp30) evidence in regulating pancreatic homeostasis and malignancies, herein we expressly review the adiponectin receptor agonist AdipoRon as both a potential anticancer agent and gemcitabine sensitizer in PDAC. Providing our personal point of view on criticism and feelings, we try to figure out whether this receptor agonist can potentially be considered a future therapeutic choice in overcoming chemotherapy-induced resistance, expressly in PDAC.

### Adiponectin: an insight on its aptitudes in regulating pancreatic homeostasis

Encoded by the *ApM1* gene (adipose most abundant gene transcript 1), Acrp30 is a 244-amino acid adipokine synthesized by adipose cells and assembled in three distinct homopolymers, which differ in molecular weight and affinity for receptors^[33]^. As one of the most abundant serum-related adipokines, Acrp30 physiologically influences a wide range of cellular functions, including either related or unrelated metabolic pathways^[34]^. Acrp30 primarily regulates glucose and fatty acids homeostasis, and supplementary beneficial properties have extensively been reported. Acrp30 can indeed mitigate pathophysiological conditions such as inflammation, atherosclerosis, and immune-mediated response^[35]^.

The pivotal role of pancreas in regulating glucose uptake makes this organ extremely vulnerable to Acrp30 signaling, even though variable and inconsistent results still exist side by side. In this respect, while many studies provide evidence supporting the Acrp30-mediated insulin release in mouse islets, in humans, this stimulation appears to be ineffective against either basal or glucose-induced insulin secretion^[36,37]^. However, there are certain considerations that could elucidate this controversy. Contrary to other adipokines, pancreas-specific Acrp30 knockout mouse models have not been engineered thus far; therefore, all the provided information merely recognizes systemic effects which could influence every single statement. Moreover, glucose levels and resistance status are two additional key issues which could contribute to Acrp30-related responses. In this connection, scrutinizing the insulin-resistant mouse islets, Winzell and colleagues warned that Acrp30 can display a dual opposite effect in stimulating this specific hypoglycemic agent, namely inhibiting insulin secretion at low glucose concentrations and promoting its release under hyperglycemic status^[38]^.

Quite convincing is the Acrp30-mediated role in protecting the maintenance and survival of functional β-cells. The available scientific research provides precise data about the anti-apoptotic properties of Acrp30 towards lipids, ceramides, and cytokines^[37,39]^. In light of its cytoprotective effects, Acrp30 has also been proposed as a potential target to treat metabolic syndrome involving β-cell dysfunction. However, the lack of compounds capable of stimulating Acrp30 production or mimicking its action has made a chimera of this therapeutic strategy.

Almost no modulations have been reported for the remaining islets components, as well as for the exocrine portion of the pancreas. Albeit β-cells constitute nearly the totality of pancreatic islets, specific unrelated changes in pancreatic functions have been detected for other adipokines. In this respect, inducing variations in membrane potential and glucagon secretion, leptin-mediated regulation has been observed in both mouse and human α-cells^[40,41]^. Alterations in lipases release were also obtained in response to leptin administration^[42]^. Instead, resistin, a putative adipocyte-derived hormone involved in insulin resistance and diabetes, significantly increased the secretion of pancreatic amylase, thus worsening inflammatory response and acute pancreatitis severity^[43]^.

Scrutinizing the scientific production, it remains quite unclear why no Acrp30 results have been achieved with respect to other pancreatic components. Surely, the limited available indirect findings suggest a chance that even these portions may be responsive to Acrp30 fluctuations. In cystic fibrosis (CF), for instance, an autosomal recessive inflammatory disorder that consistently results in pancreatic exocrine dysfunction^[44]^, Acrp30 serum levels are usually higher compared to healthy subjects^[45,46]^. Acrp30 modulations have also been reported in non-functional pancreatic cells, particularly in vascular endothelial cells (VECs), where an unusual overexpression in this adipokine may serve as a protective angiogenic factor in mice fed a high-fat diet^[47]^.

### Across the controversial relationship between adiponectin and PDAC

As recently examined in a theoretical study, the vast majority of the Acrp30-mediated functions are carried out by two highly homologous seven-transmembrane helices, termed AdipoR1 and AdipoR2, which recognized the canonical membrane receptors for this kind of adipokine^[48]^. Once recognition and binding have occurred, Acrp30 promotes the recruitment of adaptor protein APPL1, which in turn activates AMP-activated protein kinase (AMPK) and peroxisome proliferator-activated receptor α (PPARα)^[35]^. Besides regulating energy homeostasis, Acrp30-related intracellular targets are actively engaged in controlling several signaling pathways that, depending on cell specialization, may lead to opposite effects^[49]^. The third receptor in order of discovery is a calcium-dependent adhesion molecule, also referred to as T-cadherin, whereby a lack of its intracellular domain may serve as Acrp30 co-receptor for unknown signaling^[50]^.

Although the presence of Acrp30 receptors has not been confirmed in all pancreatic-derived cells yet, its involvement in PDAC initiation and progression has extensively been assessed over the years. The largest part of the existing preclinical studies designates Acrp30 as an effective anticancer molecule in PDAC models^[51,52]^. There is one piece of evidence suggesting a tissue-dependent outcome on tumorigenesis, which proposes an unconventional Acrp30 role in promoting PDAC growth^[53]^. The conflicting findings obtained by Huang and colleagues can be explained by the specific strain employed for their *in vivo* experiments. Specifically, inoculating H7 and Panc02 cells in Acrp30-KO and Acrp30-WT C57BL/6 mice, they observed that the size and weight of the resulting tumor in Acrp30-WT mice were larger compared to those achieved in Acrp30-KO. However, apart from connecting these results directly with the Acrp30 action, no accounts were taken of the potential metabolic alterations induced by its systemic ablation, which could constitute a hostile environment for tumor growth per se. However, more accurate experiments involving the use of an inducible system are mandatory to figure out the cause of this controversy.

The clinical findings supporting the Acrp30 involvement in pancreatic malignancy as a putative risk and prognostic factor remain inconclusive. While both US and European nested case-control studies revealed an inverse correlation between the pre-diagnostic plasma levels of Acrp30 and the subsequent risk of developing PDAC^[54,55]^, a recent Mendelian randomized analysis totally rejects this association^[56]^. Two main limitations make the link between Acrp30 and PDAC risk inconclusive so far: (i) the absence of systematic reviews of randomized controlled trials; and (ii) the limited setting of PDAC patients whose Acrp30 levels were monitored before and during PDAC diagnosis. Moreover, rather than an ordinary relationship with blood levels, PDAC risk could be associated with the expression of specific genetic variants of Acrp30^[57]^. More persuasive appears the hyperadiponectinemia observed in PDAC instead^[58-60]^. While this connection could be interpreted as a compensatory response to the PDAC-induced cachexia, Acrp30 plasma levels seem unrelated to either BMI or leptin concentration^[58,59]^. Therefore, an alternative explanation for this unexpected increase could be a sort of adaptive process for hindering tumor growth. This model could also account for the reduced levels of both Acrp30 receptors observed in PDAC cells relative to normal pancreatic tissue^[61]^. Nevertheless, further designs are absolutely needed to investigate the above, as well as other possible conjectures. Only few and conflicting assumptions have been stated regarding Acrp30 as a prognostic factor in PDAC^[62,63]^.

### AdipoRon and PDAC: more lights than shadows

Due to a combination of both intrinsic and extrinsic limitations, no translation has been conceived for Acrp30 in cancer clinical trials. While the controversial findings have not permitted any subsequent steps involving PDAC patients to date, Acrp30 translation floundered even in cancers showing very convincing preclinical results. The heavy molecular mass and the reduced half-life/stability of Acrp30 have substantially hampered any clinical application in malignancy as well as in other pathological conditions. Nevertheless, the recent characterization of a plausible Acrp30 active and binding site has broken new ground in the design of derived compounds, thus making different druggable options available^[64]^.

In regard to the Acrp30-mediated beneficial properties observed in cell disease models, adiponectin receptor agonists certainly denote the most promising class of compounds having potential therapeutic perspectives. Among the other agonists, AdipoRon is emerging as a fascinating anticancer agent in several malignancies, including osteosarcoma, myeloma, breast and ovarian cancer^[65,66]^. Standing out as the top-scored in activating AMPK and bonding AdipoR1 and AdipoR2 in murine myeloblast C2C12 cells, AdipoRon is currently recognized as the first orally active adiponectin receptor agonist^[67]^.

Independent research groups have provided convincing evidence supporting the antiproliferative role played by AdipoRon in both *in vitro* and *in vivo* PDAC models^[61,68-71]^. Unsurprisingly, querying the main scientific databases for AdipoRon, PDAC represents the most mentioned among the tumor models examined thus far. A detailed summary of the key findings obtained in PDAC is discussed hereafter.

Messaggio and co-workers were the first to prove that AdipoRon could suppress tumor growth in PDAC models^[61]^. Specifically, employing both human and GEMM-derived mouse PDAC cells, they observed reduced proliferation and apoptosis induction in response to AdipoRon administration concurrently. Even though AdipoRon treatments decreased signal transducer and activator of transcription 3 (STAT3) phosphorylation, no experiments have been performed to address its impact on the AdipoRon-mediated action. In this respect, a more comprehensive mechanistic design was proposed by Akimoto and colleagues a year later, who drew attention to RIPK1-dependent necroptosis as the main tool for cell death induction in AdipoRon-treated cells^[68]^. Nevertheless, based on the obtained results, they did not exclude that caspase-independent apoptosis and autophagy could be similarly involved. Mechanistically, a rise in calcium concentration triggered extracellular signal-regulated protein kinase 1/2 (ERK1/2) and calpain-1 activation, which in turn led to mitochondrial dysfunction and caspase-independent apoptosis induction. Activation of AMPK and p38 signaling were recognized as survival pathways in this specific experimental design, since their pharmacological inhibition enhanced the AdipoRon cytotoxic effects. The impairment of mitochondrial activity was further confirmed by Manley *et al*. in their latest study^[69]^. Performing Seahorse-based assays, they noticed that AdipoRon treatments not only decreased basal and maximal mitochondrial respiration but also attenuated proton leak. As a compensatory response to defective mitochondrial ATP production, PDAC cells increased anaerobic glycolysis by consuming a greater amount of glucose and producing more lactic acid. Finally, based on these results, the authors recognized glycolysis inhibitors as potential targets to enhance AdipoRon effectiveness. Aimed at providing more insight into the AdipoRon-mediated antitumor properties in PDAC, Takenaga and colleagues revealed a concurrent angiogenesis inhibition in MSS31 endothelial cells, which may cause a shortage of nutrients and oxygen supply in tumor cells^[71]^. Although AMPK, p38, and ERK1/2 were simultaneously activated by AdipoRon in this cell type, the MEK1 inhibitor U0126 was the only one capable of preserving tube formation. The anti-angiogenic features could also explain the reduced effectiveness observed in the AdipoRon-treated orthotopic pancreatic cancer mice fed a high-fat diet. Specifically, given that obese mice usually show high plasma levels of leptin, this anorexigenic adipokine may dynamically compete with AdipoRon in stimulating endothelial cells, thus overriding pro- rather than anti-angiogenic mechanisms.

### AdipoRon improves gemcitabine-mediated outcomes in PDAC cells

While different studies convincingly propose AdipoRon as an anticancer agent in PDAC models, no data support a potential cooperating effect in gemcitabine-based therapy. In this respect, we recently provided evidence of increased responsiveness to gemcitabine when PDAC cells were concomitantly stimulated with AdipoRon. Using multiple biological approaches, we demonstrated that AdipoRon plus gemcitabine significantly decreased tumorigenesis in two distinct PDAC cell lines, MIA PaCa-2 and PANC-1.

In detail, adding AdipoRon to gemcitabine further compromised cell growth and colony-forming ability compared with single treatments, thus suggesting a positive correlation between these two compounds. For this purpose, CompuSyn analysis was subsequently performed, demonstrating a potential synergistic effect in either raising cell-growth inhibition or reducing colony formation. Precisely, this assay revealed a combination index (CI) ranging from 0.59-0.63 in MIA PaCa-2 and 0.05-0.22 in PANC-1. We also recorded G0/G1 and S phase accumulation after the individual release of AdipoRon and gemcitabine, respectively. Remarkably, when these two compounds were administrated together, we noticed different but intermediate features in cell cycle distribution. Without statistically significant variations in SubG1 amount compared to the deoxycytidine analog (*P* > 0.05), AdipoRon plus gemcitabine induced G0/G1 accumulation closer to AdipoRon within 24 h and S-phase arrest similar to gemcitabine after 48 h. The observed variations in cell cycle distribution after AdipoRon, gemcitabine, and AdipoRon plus gemcitabine treatment were further corroborated by cyclins and cyclin-dependent kinase inhibitor levels. Considering the relevance of the ERK1/2 signaling in the AdipoRon-mediated action, we investigated its possible involvement in combination with gemcitabine. Without significant changes in total protein amount, we observed a phospho-ERK1/2 upregulation in combined treatment more than AdipoRon alone. Intriguingly, the usage of PD98059 as a potent MEK1/MEK2 inhibitor partially prevented the enhanced outcomes observed in reaction to AdipoRon plus gemcitabine, thus reinforcing the dynamic influence of this pathway in the AdipoRon-mediated sensitization. To further speculate on the usefulness of AdipoRon-based therapy in PDAC, we finally explored the combination impact in gemcitabine-resistant MIA PaCa-2 cells. While gemcitabine was ineffective in decreasing cell proliferation, AdipoRon treatments, either alone or in combination, hindered cell growth with an inhibition rate of 25% and 43%, respectively. We also observed a reduction in colony numbers after both AdipoRon and AdipoRon plus gemcitabine administration. As in gemcitabine-sensitive cells, AdipoRon was able to increase the G0/G1 phase in resistant ones, while the simultaneous presence of gemcitabine further intensified this tendency.

### Critical issues and future perspectives for the AdipoRon usage in PDAC treatment

In light of the latest cancer statistics^[3]^, it is explicit that all clinically approved therapeutic strategies for treating PDAC have provided unsatisfactory responses. While the rest of the cancers are moving toward a chronic management, PDAC remains one of the deadliest malignancies worldwide^[72]^. Even immunotherapy, which has been designed as the “breakthrough” in cancer treatment, has achieved only weak outcomes in PDAC^[73]^. Consequently, identifying novel pharmacological approaches is categorically demanded to make PDAC more treatable.

AdipoRon has recently been found to be effective in contrasting PDAC cell growth at preclinical stage^[61,68,69,71]^, but, more interestingly, we recently provided evidence of potential cooperating effects between AdipoRon and gemcitabine^[70]^. Due to the low rate of radiation and surgery eligibility, gemcitabine-based therapy constitutes the widely used approach in treating PDAC^[5]^. Regrettably, even though gemcitabine is still considered a cornerstone in PDAC therapy, the chances of developing chemoresistance are extremely high for these patients, and thereby combinatory treatments are usually preferred over single-agent administration. Despite the huge efforts made to provide other pharmacological options, only two gemcitabine partners, erlotinib and nab-paclitaxel, have been approved in clinic^[74]^. However, their success rate is strictly dependent on several factors, including genetic signatures and performance status scale^[75,76]^. Recognizing AdipoRon as a potential candidate in gemcitabine-based therapy may provide additional hopes for advanced PDAC patients. However, to be fair, the possibility that AdipoRon may reach the bedside of PDAC patients is currently remote. While AdipoRon has already been tested in animal models as a single agent, showing a high degree of tolerability in normal tissues^[68]^, *in vivo* studies have not corroborated its effectiveness in combination with gemcitabine yet. Recently, another adiponectin receptor agonist has concluded phase 1/2a (NCT04201574) and is currently under evaluation in phase 2/3 clinical trials (NCT04899518). As a peptidomimetic agonist, ALY688 has shown no specific adverse reactions as ophthalmic solution in human patients, and now two distinct concentrations (0.4% and 1%) will be evaluated for their efficacy in subjects with dry eye disease. While extremely promising, a positive ALY688 outcome in human trials would not lead to AdipoRon approval in clinic, but it could pose a driving force for its testing in cancer patients. Adiponectin receptor agonists actually include a heterogeneous group of molecules, and thus they must be investigated individually at every stage. In this respect, dissimilarities in biological behaviors have been observed even between AdipoRon and Acrp30. Characterizing the AdipoRon-mediated mechanism of action in PDAC cells, Akimoto and colleagues noticed that, unlike AdipoRon, neither ERK1/2 activation nor cell death induction was detected in response to Acrp30 administration^[68]^. These findings bring more doubts about the chemical and biological differences that may exist between these two molecules, as well as whether the AdipoRon-mediated effects are really due to the agonistic action towards Acrp30 receptors.

The ability to make gemcitabine-resistant MIA PaCa-2 cells responsive to AdipoRon denotes a fascinating aptitude that could open up new opportunities in overcoming chemotherapy refractoriness. On the basis of the standing knowledge, there is a rational explanation for corroborating the above biological outcome. Recently, mitochondria have been described to facilitate the survival of stem and dormant cells treated with cytotoxic agents in PDAC^[31]^. Several mitochondrial-related pathways and mechanisms have been associated with chemoresistance, such as apoptosis, autophagy, and metabolic remodeling^[77]^. In accordance with this latter operation, in both oncogene-ablated and gemcitabine-treated PDAC cells, targeting OXPHOS significantly shrank tumor recurrence^[31,32,78]^. Since an OXPHOS impairment has also been observed in response to AdipoRon administration^[68,69]^, it is plausible to imagine a link between the damage that occurred at this organelle and the ability to sensitize PDAC resistant cells to gemcitabine. While the aberrant glycolysis usage is considered a metabolic feature of drug resistance in tumor cells^[30,79]^, after AdipoRon stimulation, this compensatory mechanism could be exploited to completely eradicate the minimal residual disease [Table 1]^[69,80,81]^. Besides the metabolic outlook, another potential mechanistic way to corroborate the AdipoRon-mediated gemcitabine sensitization could be represented by the ABC transporters, which have recently been implicated in chemotherapy resistance, expressly in PDAC^[21-23]^. Despite no proof currently demonstrating an AdipoRon-induced ABCs modulation, limited findings correlate Acrp30 and some members of this class of transporters. In this respect, Acrp30 has been described to increase both mRNA and protein levels of ABCA1 in hepatocellular carcinoma HepG2 cells^[82]^. A positive correlation between Acrp30 and ABCA1 levels has also been observed in visceral adipose tissue^[83]^. However, the abovementioned speculation first warrants further investigation about the possible contribution of ABC transporters to gemcitabine resistance.

**Table 1:** Metabolic pathways involved in gemcitabine resistance and potentially affected by AdipoRon exposure

**Metabolic pathway**	**Molecular target**	**Drug inhibitor**	**Reference**
OXPHOS	Complex I	Metformin, rotenone, phenformin	[31]
	Complex IV	Arsenic trioxide	[32]
	Complex V	Oligomycin	[78]
Glycolysis	Hexokinase	2-deoxy-D-glucose	[79]
	Pyruvate Dehydrogenase	CPI-613 (devimistat)	[32]
	LDH-A	N-hydroxyindole-based inhibitors	[80,81]

**Figure 1:**
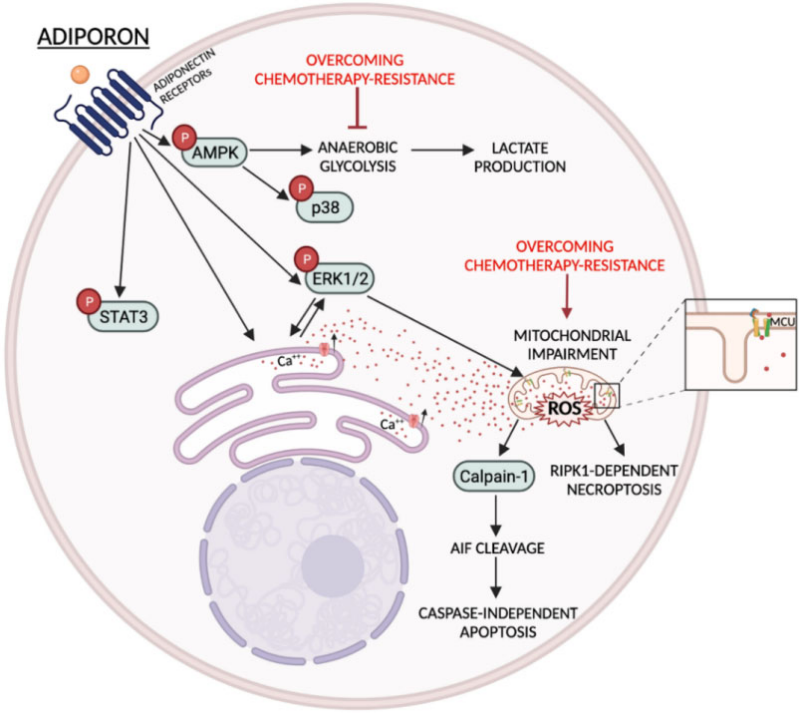
AdipoRon-mediated mechanisms and potential interconnection points to overcome gemcitabine resistance in PDAC. Schematic representation of the main metabolic and signaling pathways regulated by AdipoRon in PDAC cells. Red arrows indicate direct and indirect AdipoRon-related ways to hinder chemotherapy resistance.

Although all these assumptions are largely based on logical reasoning, we strongly feel that all future inquiries should be moving in this direction. In addition, the next two steps should be aimed at defining the existence of potential cooperating effects with other approved chemo-drugs and translating the combination of AdipoRon plus gemcitabine into a more complex biological system such as *in vivo* models. Simultaneously, characterizing each mechanistic aspect of the AdipoRon-mediated features may contribute to outlining its wholesomeness in overcoming gemcitabine and, more in general, chemotherapy-induced resistance in PDAC [Figure 1].

In light of the current orphan status and the tremendously unfavorable prognosis, each promising molecule capable of improving both PDAC prognosis and survival should be fully explored. Analyzing the current scientific production, AdipoRon is emerging as a potential therapeutic choice in PDAC, either as a single compound or a partner in gemcitabine-based treatment. Therefore, its pharmacological properties perfectly fulfill the requirements that a candidate is supposed to have for ameliorating PDAC expectation. Whether this is enough to attain clinical approval will be based on the upcoming experiments. 

## DECLARATIONS

### Copyright

© The Author(s) 2022.

## References

[1] Park W, Chawla A, O'Reilly EM (2021). Pancreatic cancer: a review. JAMA.

[2] Sridharan V, Hernandez-Barco YG, Ting DTJAoPC

[3] Siegel RL, Miller KD, Fuchs HE, Jemal A (2021). Cancer statistics, 2021. CA Cancer J Clin.

[4] Palmeri M, Di Franco G, Bianchini M (2021). Prognostic impact of conservative surgery for pancreatic IPMNs. Surg Oncol.

[5] Müller PC, Frey MC, Ruzza CM (2021). Neoadjuvant chemotherapy in pancreatic cancer: an appraisal of the current high-level evidence. Pharmacology.

[6] Conroy T, Hammel P, Hebbar M, Canadian Cancer Trials Group and the Unicancer-GI-PRODIGE Group (2018). FOLFIRINOX or gemcitabine as adjuvant therapy for pancreatic cancer. N Engl J Med.

[7] Mehta A, Hwang WL, Weekes C (2020). The present and future of systemic and microenvironment-targeted therapy for pancreatic adenocarcinoma. Ann Pancreat Cancer.

[8] Yu S, Zhang C, Xie KP (2021). Therapeutic resistance of pancreatic cancer: roadmap to its reversal. Biochim Biophys Acta Rev Cancer.

[9] Tuerhong A, Xu J, Shi S (2021). Overcoming chemoresistance by targeting reprogrammed metabolism: the Achilles’ heel of pancreatic ductal adenocarcinoma. Cell Mol Life Sci.

[10] Randazzo O, Cascioferro SM, Pecoraro C (2021). SF3B1 modulators affect key genes in metastasis and drug influx: a new approach to fight pancreatic cancer chemoresistance. Cancer Drug Resist.

[11] Aughton K, Elander NO, Evans A (2021). hENT1 predicts benefit from gemcitabine in pancreatic cancer but only with low CDA mRNA. Cancers (Basel).

[12] Du J, Gu J, Li J (2020). Mechanisms of drug resistance of pancreatic ductal adenocarcinoma at different levels. Biosci Rep.

[13] Saiki Y, Hirota S, Horii A (2020). Attempts to remodel the pathways of gemcitabine metabolism: recent approaches to overcoming tumours with acquired chemoresistance. Cancer Drug Resist.

[14] Yi-zheng X, Plunkett W (1992). Modulation of deoxycytidylate deaminase in intact human leukemia cells. Biochemical Pharmacology.

[15] Heinemann V, Xu YZ, Chubb S et al (1992). Cellular elimination of 2',2'-difluorodeoxycytidine 5'-triphosphate: a mechanism of self-potentiation. Cancer Res.

[16] Serdjebi C, Seitz JF, Ciccolini J (2013). Rapid deaminator status is associated with poor clinical outcome in pancreatic cancer patients treated with a gemcitabine-based regimen. Pharmacogenomics.

[17] Miller AL, Garcia PL, Gamblin TL, Vance RB, Yoon KJ (2020). Development of gemcitabine-resistant patient-derived xenograft models of pancreatic ductal adenocarcinoma. Cancer Drug Resist.

[18] Weizman N, Krelin Y, Shabtay-Orbach A (2014). Macrophages mediate gemcitabine resistance of pancreatic adenocarcinoma by upregulating cytidine deaminase. Oncogene.

[19] Xiao H, Zheng Y, Ma L, Tian L, Sun Q (2021). Clinically-relevant ABC transporter for anti-cancer drug resistance. Front Pharmacol.

[20] Gupta SK, Singh P, Ali V, Verma M (2020). Role of membrane-embedded drug efflux ABC transporters in the cancer chemotherapy. Oncol Rev.

[21] Xu M, Li L, Liu Z (2013). ABCB2 (TAP1) as the downstream target of SHH signaling enhances pancreatic ductal adenocarcinoma drug resistance. Cancer Lett.

[22] Lu Y, Xu D, Peng J (2019). HNF1A inhibition induces the resistance of pancreatic cancer cells to gemcitabine by targeting ABCB1. EBioMedicine.

[23] Okada Y, Takahashi N, Takayama T, Goel A (2021). LAMC2 promotes cancer progression and gemcitabine resistance through modulation of EMT and ATP-binding cassette transporters in pancreatic ductal adenocarcinoma. Carcinogenesis.

[24] Boyd LNC, Andini KD, Peters GJ, Kazemier G, Giovannetti E (2022). Heterogeneity and plasticity of cancer-associated fibroblasts in the pancreatic tumor microenvironment. Semin Cancer Biol.

[25] Jiang B, Zhou L, Lu J (2020). Stroma-targeting therapy in pancreatic cancer: one coin with two sides?. Front Oncol.

[26] Li MJAoPC (2020). Preface for focused issue: science on pancreatic cancer. Ann Pancreat Cancer.

[27] Ni Y, Zhou X, Yang J (2021). The role of tumor-stroma interactions in drug resistance within tumor microenvironment. Front Cell Dev Biol.

[28] Comandatore A, Immordino B, Balsano R (2022). Potential role of exosomes in the chemoresistance to gemcitabine and nab-paclitaxel in pancreatic cancer. Diagnostics (Basel).

[29] Grasso C, Jansen G, Giovannetti E (2017). Drug resistance in pancreatic cancer: impact of altered energy metabolism. Crit Rev Oncol Hematol.

[30] Dai S, Peng Y, Zhu Y (2020). Glycolysis promotes the progression of pancreatic cancer and reduces cancer cell sensitivity to gemcitabine. Biomed Pharmacother.

[31] Masoud R, Reyes-Castellanos G, Lac S (2020). Targeting mitochondrial complex I overcomes chemoresistance in high OXPHOS pancreatic cancer. Cell Rep Med.

[32] Reyes-Castellanos G, Masoud R, Carrier A (2020). Mitochondrial metabolism in PDAC: from better knowledge to new targeting strategies. Biomedicines.

[33] Khoramipour K, Chamari K, Hekmatikar AA (2021). Adiponectin: structure, physiological functions, role in diseases, and effects of nutrition. Nutrients.

[34] Esmaili S, Hemmati M, Karamian M (2020). Physiological role of adiponectin in different tissues: a review. Arch Physiol Biochem.

[35] Choi HM, Doss HM, Kim KS (2020). Multifaceted physiological roles of adiponectin in inflammation and diseases. Int J Mol Sci.

[36] Lee YH, Magkos F, Mantzoros CS, Kang ES (2011). Effects of leptin and adiponectin on pancreatic β-cell function. Metabolism.

[37] Tao C, Sifuentes A, Holland WL (2014). Regulation of glucose and lipid homeostasis by adiponectin: effects on hepatocytes, pancreatic β cells and adipocytes. Best Pract Res Clin Endocrinol Metab.

[38] Winzell MS, Nogueiras R, Dieguez C, Ahrén B (2004). Dual action of adiponectin on insulin secretion in insulin-resistant mice. Biochem Biophys Res Commun.

[39] Bakhti M, Lickert H (2022). New insights into β-cell failure, regeneration and replacement. Nat Rev Endocrinol.

[40] Tudurí E, Marroquí L, Soriano S (2009). Inhibitory effects of leptin on pancreatic alpha-cell function. Diabetes.

[41] Tudurí E, Denroche HC, Kara JA, Asadi A, Fox JK, Kieffer TJ (2014). Partial ablation of leptin signaling in mouse pancreatic α-cells does not alter either glucose or lipid homeostasis. Am J Physiol Endocrinol Metab.

[42] Elinson N, Amichay D, Birk RZ (2006). Leptin directly regulates exocrine pancreas lipase and two related proteins in the rat. Br J Nutr.

[43] Jiang CY, Wang W (2017). Resistin aggravates the expression of proinflammatory cytokines in cerulein-stimulated AR42J pancreatic acinar cells. Mol Med Rep.

[44] Ramkissoon R, Gardner TB (2019). Pancreatic steatosis: an update. Curr Opin Gastroenterol.

[45] Panagopoulou P, Fotoulaki M, Manolitsas A, Pavlitou-Tsiontsi E, Tsitouridis I, Nousia-Arvanitakis S (2008). Adiponectin and body composition in cystic fibrosis. J Cyst Fibros.

[46] Polito R, Nigro E, Elce A (2019). Adiponectin expression is modulated by long-term physical activity in adult patients affected by cystic fibrosis. Mediators Inflamm.

[47] Liu XX, Liu KY, Li P, Han S, Peng XD, Shen L (2014). Adiponectin is expressed in the pancreas of high-fat-diet-fed mice and protects pancreatic endothelial function during the development of type 2 diabetes. Diabetes Metab.

[48] Muratore M, Komai AM (2020). Theoretical study of the adiponectin receptors: binding site characterization and molecular dynamics of possible ligands for drug design. SN Appl Sci.

[49] Roy B, Palaniyandi SS (2021). Tissue-specific role and associated downstream signaling pathways of adiponectin. Cell Biosci.

[50] Rubina KA, Semina EV, Kalinina NI, Sysoeva VY, Balatskiy AV, Tkachuk VA (2021). Revisiting the multiple roles of T-cadherin in health and disease. Eur J Cell Biol.

[51] Jiang J, Fan Y, Zhang W (2019). Adiponectin suppresses human pancreatic cancer growth through attenuating the β-catenin signaling pathway. Int J Biol Sci.

[52] Kato M, Watabe K, Tsujii M, Funahashi T, Shimomura I, Takehara T (2014). Adiponectin inhibits murine pancreatic cancer growth. Dig Dis Sci.

[53] Huang B, Cheng X, Wang D (2014). Adiponectin promotes pancreatic cancer progression by inhibiting apoptosis via the activation of AMPK/Sirt1/PGC-1α signaling. Oncotarget.

[54] Bao Y, Giovannucci EL, Kraft P (2013). A prospective study of plasma adiponectin and pancreatic cancer risk in five US cohorts. J Natl Cancer Inst.

[55] Grote VA, Rohrmann S, Dossus L (2012). The association of circulating adiponectin levels with pancreatic cancer risk: a study within the prospective EPIC cohort. Int J Cancer.

[56] Dimou NL, Papadimitriou N, Mariosa D, CCFR, Endometrial Cancer Association Consortium (2021). Circulating adipokine concentrations and risk of five obesity-related cancers: a mendelian randomization study. Int J Cancer.

[57] Yang JP, Li X, Wang F, Gao M, Li SL, Chen KS (2015). Association analysis of genetic variants of adiponectin gene and risk of pancreatic cancer. Int J Clin Exp Med.

[58] Dalamaga M, Migdalis I, Fargnoli JL (2009). Pancreatic cancer expresses adiponectin receptors and is associated with hypoleptinemia and hyperadiponectinemia: a case-control study. Cancer Causes Control.

[59] Dranka-Bojarowska D, Lekstan A, Olakowski M et al (2015). The assessment of serum concentration of adiponectin, leptin and serum carbohydrate antigen-19.9 in patients with pancreatic cancer and chronic pancreatitis. J Physiol Pharmaco.

[60] Eibl G, Rozengurt E (2021). Obesity and pancreatic cancer: insight into mechanisms. Cancers (Basel).

[61] Messaggio F, Mendonsa AM, Castellanos J (2017). Adiponectin receptor agonists inhibit leptin induced pSTAT3 and in vivo pancreatic tumor growth. Oncotarget.

[62] Kadri Colakoglu M, Bostanci EB, Ozdemir Y (2017). Roles of adiponectin and leptin as diagnostic markers in pancreatic cancer. Bratisl Lek Listy.

[63] Zyromski NJ, Mathur A, Pitt HA (2009). Obesity potentiates the growth and dissemination of pancreatic cancer. Surgery.

[64] Otvos L Jr (2019). Potential adiponectin receptor response modifier therapeutics. Front Endocrinol (Lausanne).

[65] Nigro E, Daniele A, Salzillo A, Ragone A, Naviglio S, Sapio L (2021). AdipoRon and other adiponectin receptor agonists as potential candidates in cancer treatments. Int J Mol Sci.

[66] Sapio L, Nigro E, Ragone A (2020). AdipoRon affects cell cycle progression and inhibits proliferation in human osteosarcoma cells. J Oncol.

[67] Okada-Iwabu M, Yamauchi T, Iwabu M (2013). A small-molecule AdipoR agonist for type 2 diabetes and short life in obesity. Nature.

[68] Akimoto M, Maruyama R, Kawabata Y, Tajima Y, Takenaga K (2018). Antidiabetic adiponectin receptor agonist AdipoRon suppresses tumour growth of pancreatic cancer by inducing RIPK1/ERK-dependent necroptosis. Cell Death Dis.

[69] Manley SJ, Olou AA, Jack JL (2022). Synthetic adiponectin-receptor agonist, AdipoRon, induces glycolytic dependence in pancreatic cancer cells. Cell Death Dis.

[70] Ragone A, Salzillo A, Spina A, Naviglio S, Sapio L (2022). Integrating gemcitabine-based therapy with adiporon enhances growth inhibition in human PDAC cell lines. Front Pharmacol.

[71] Takenaga K, Akimoto M, Koshikawa N, Nagase H (2021). Obesity reduces the anticancer effect of AdipoRon against orthotopic pancreatic cancer in diet-induced obese mice. Sci Rep.

[72] Latenstein AEJ, van der Geest LGM, Bonsing BA, Dutch Pancreatic Cancer Group (2020). Nationwide trends in incidence, treatment and survival of pancreatic ductal adenocarcinoma. Eur J Cancer.

[73] Mucciolo G, Roux C, Scagliotti A, Brugiapaglia S, Novelli F, Cappello P (2020). The dark side of immunotherapy: pancreatic cancer. Cancer Drug Resist.

[74] Elsayed M, Abdelrahim M (2021). The latest advancement in pancreatic ductal adenocarcinoma therapy: a review article for the latest guidelines and novel therapies. Biomedicines.

[75] Blomstrand H, Scheibling U, Bratthäll C, Green H, Elander NO (2019). Real world evidence on gemcitabine and nab-paclitaxel combination chemotherapy in advanced pancreatic cancer. BMC Cancer.

[76] Hoyer K, Hablesreiter R, Inoue Y (2021). A genetically defined signature of responsiveness to erlotinib in early-stage pancreatic cancer patients: results from the CONKO-005 trial. EBioMedicine.

[77] Santofimia-Castaño P, Iovanna J (2021). Combating pancreatic cancer chemoresistance by triggering multiple cell death pathways. Pancreatology.

[78] Viale A, Pettazzoni P, Lyssiotis CA (2014). Oncogene ablation-resistant pancreatic cancer cells depend on mitochondrial function. Nature.

[79] Peng J, Cui Y, Xu S (2021). Altered glycolysis results in drug-resistant in clinical tumor therapy. Oncol Lett.

[80] Li Petri G, El Hassouni B, Sciarrillo R (2020). Impact of hypoxia on chemoresistance of mesothelioma mediated by the proton-coupled folate transporter, and preclinical activity of new anti-LDH-A compounds. Br J Cancer.

[81] Hassouni BE, Franczak M, Capula M (2020). Lactate dehydrogenase A inhibition by small molecular entities: steps in the right direction. Oncoscience.

[82] Matsuura F, Oku H, Koseki M (2007). Adiponectin accelerates reverse cholesterol transport by increasing high density lipoprotein assembly in the liver. Biochem Biophys Res Commun.

[83] Vincent V, Thakkar H, Aggarwal S, Mridha A, Ramakrishnan L, Singh A

